# *Amanita bisporigera*-Induced Hepatic Failure: A Fatal Case of Mushroom Ingestion

**DOI:** 10.1155/2011/936867

**Published:** 2011-12-18

**Authors:** Anthony Nici, Sang Kim

**Affiliations:** New York Hospital Queens, Weill Medical College of Cornell University, 56-45 Main Street, Flushing, NY 11355, USA

## Abstract

Wild mushroom poisoning from the genus Amanita is a medical emergency, with Amanita phalloides being the most common offender. Patients may complain of nausea, vomiting, diarrhea and/or abdominal pain. If not aggressively treated, fulminant hepatic failure may develop within several days of ingestion. In this case report, a patient poisoned with *Amanita bisporigera* is described, along with the typical clinical presentation, patient outcomes, and treatment options for dealing with an Amanita mushroom poisoning.

## 1. Introduction

Wild mushroom poisoning cases leading to fatal liver-induced injury usually are attributed to* Amanita phalloides* ingestion in the United States. However an infrequently documented mushroom species *Amanita bisporigera* has also been found to be just as deadly in producing acute fulminant liver failure. The following is a reported case of such an occurrence. 

## 2. Case Report

We report the case of a 53-year-old female who presented to the emergency room complaining of severe nausea and vomiting associated with abdominal cramps and intense diarrhea starting nine hours after ingesting wild mushrooms which she had picked herself. The patient was admitted to the hospital and started on an intense cocktail of therapeutic modalities. The patient reported that at 4 am she awoke with intense right upper quadrant (RUQ) abdominal pain, accompanied by severe nausea and innumerable amounts of emesis. In addition she reported having multiple episodes of watery diarrhea. The symptoms continued throughout the day until she arrived in the emergency room. In the emergency room she was noticed to be in no acute distress with a temperature of 97.3 degrees Fahrenheit, blood pressure of 153/84, pulse of 96, and a respiratory rate of 20. Her physical examination was notable for an abdominal examination significant for mild tenderness to palpation in the RUQ with no guarding or rebound, active bowel sounds, or stigmata of liver disease. Pertinent laboratory values on admission revealed a creatinine of 1.2 mg/dL, WBC of 14.2 K/uL, hemoglobin of 16.7 g/dL, hematocrit of 49.3%, INR of 1.1, total bilirubin 0.8 mg/dL, ALT of 54 u/L, and AST of 68 u/L.

 Intravenous hydration with normal saline was started and the New York State Poison Control Center was contacted. They recommended administering a regimen consisting of oral-activated charcoal 50 g every 2 hours, penicillin G one million units/kg IVPB every 6 hours divided into four doses per day, famotidine 20 mg IVPB every 6 hours (used for the first 16 hours of care until cimetidine 300 mg IV could be acquired), and vitamin C 500 mg PO BID. The following morning the patient's blood tests showed an increased ALT of 223 u/L and an AST of 304 u/L. She reported that she no longer had anymore nausea, vomiting, diarrhea, or abdominal cramps and that she was feeling better. However early that afternoon the patient suddenly became hypotensive and obtunded. An arterial blood gas was obtained and revealed a severe anion gap metabolic acidosis with a pH of 7.13, PO2 113, PCO2 19.7, HCO3 6.5, and an SO2 of 92%. The patient was immediately intubated and transferred to the intensive care unit where vasopressors were started as well as sodium bicarbonate and N-acetylcysteine infusions. Repeat labs showed an ALT of 824 u/L, AST of 1258 u/L, and an INR of 2.1. At this point arrangements were immediately made to transfer the patient to our regional liver transplant center to be evaluated for a liver transplant as her condition was rapidly worsening. Upon reaching the transplant center her condition continued to degrade and within 24 hours developed fulminant liver failure accompanied by hepato-renal syndrome and died despite maximal supportive care. The culprit mushrooms that were cooked and ingested by the patient were obtained for analysis as well as any mushrooms remaining from her reported area of foraging. These samples were then examined by an expert mycologist and determined them to be of the species *Amanita bisporigera*.

## 3. Discussion

The species *Amanita bisporigera* is a lesser known cause of amanita mushroom-related deaths in North America, as most deaths are usually related to the species *Amanita phalloides*. *Amanita bisporigera*, also known as the “Destroying Angel”, is one of nine amatoxin containing mushroom species from the genus *Amanita*. According to the North American Mycological Association a total of four deaths due to *Amanita bisporigera* have been reported over the past 30 years. In a 2006 statistical report by the American Association of Poison Control Centers, 63 amatoxin poisoning cases were reported, with only two deaths [1]. Amanita mushrooms can be identified by a ring on their stem as well as a bulb called the volva which is located at the stem base. These features can easily be missed and confused for an edible mushroom to an untrained person. It is also extremely hard for any nonmushroom expert to distinguish between the multiple amanita species such as *Amanita phalloides* and *Amanita bisporigera *where small nuances in phenotype can segregate species.

 When suspecting an amanita mushroom toxin ingestion it is essential to know the duration of time between the first ingestion and eventual hospital admission since rapid hepatic decompensation can occur despite maximal supportive therapy. Amatoxin poisoning is characterized initially by an asymptomatic delay (8–14 hours), followed by the violent onset of gastrointestinal symptoms (persisting for 1-2 days), and progressive signs of hepatic damage (36–48 hours) which can lead to acute hepatic failure, coma, and death (6–16 days) [2]. All symptomatic and asymptomatic individuals who have possibly consumed these mushrooms should be immediately evaluated and prophylactically treated to prevent toxin absorption [3]. The local poison control center should be immediately contacted and intravenous fluid hydration started as soon as possible to treat any intravascular volume depletion from the initial vomiting and profuse diarrhea episodes. Since there is no effective antidote, detoxification measures should be undertaken with nasogastric tube-directed activated charcoal to bind and prevent the constant enterohepatic circulation which is the mechanism by which the amatoxin poison causes such devastating destruction to the hepatocytes of the liver.

The only recommended therapeutic options discussed in the literature are supported by anecdotal case reports and animal subject trials. It is felt that the administration of intravenous benzyl penicillin should be started in any suspected amatoxin mushroom ingestion case, which will compete with the amatoxin for binding sites on serum proteins and may even diminish hepatocellular toxin uptake. In addition to benzyl penicillin administration intravenous silybin dihemisuccinate is recommended (an extract of milk thistle which is not available for usage in the United States) which may further interrupt the enterohepatic circulation of the amatoxin pathway. The administration of silybin is thought to inhibit the binding of amatoxin to hepatocytes and compete with amatoxin for transmembrane transport. Other suggested treatment strategies include the potential antioxidant properties of ascorbic acid, cimetidine, and N-acetylcysteine (NAC). Ascorbic acid inhibits lipid peroxidation and has been used as a hepatocyte protector in acetaminophen and CCL4 poisoning/toxicity [4]. Intravenous cimetidine usage in amatoxin poisoning cases is based on its inhibition of the hepatic cytochrome P450 system, due to the clinical similarity of amanita poisoning to other toxins that affect the same pathway. Finally N-acetylcysteine's potential therapeutic effect is that it acts as a glutathione precursor when the body's natural stores are depleted making it capable of binding amatoxin-related free radicals. Its use in this setting is extrapolated from its effectiveness in treating acetaminophen poisoning.

Finally serial monitoring for liver function decompensation with attentive foresight for transferring the patient to a liver transplant center should be constantly reevaluated for any suspected or confirmed amanita mushroom ingestion case. This is important to document as a time interval of less than eight hours between mushroom ingestion and the onset of diarrhea symptoms is associated with an increased mortality [5]. This however is only a recommendation and not a rule as our index case despite having a nine-hour time interval from mushroom ingestion to symptom onset still developed fulminant liver failure and died. One way to help prevent future amatoxin-related deaths from occurring is to educate the community on the dangers of wild mushroom foraging and by increasing awareness in the medical community regarding early diagnosis and aggressive treatment regimens.
